# Impact of the COVID-19 Pandemic on Human Papillomavirus Vaccination in Brazil

**DOI:** 10.3389/ijph.2022.1604224

**Published:** 2022-03-31

**Authors:** Tércia Moreira Ribeiro Da Silva, Ana Carolina Micheletti Gomide Nogueira de Sá, Mark Anthony Beinner, Mery Natali Silva Abreu, Fernanda Penido Matozinhos, Ana Paula Sayuri Sato, Ed Wilson Rodrigues Vieira

**Affiliations:** ^1^ Department of Maternal and Child Nursing and Public Health, School of Nursing, Universidade Federal de Minas Gerais, Belo Horizonte, Brazil; ^2^ School of Nursing, Federal University of Minas Gerais, Belo Horizonte, Brazil; ^3^ School of Public Health, University of São Paulo, São Paulo, Brazil

**Keywords:** epidemiology, immunization programs, adolescent health, immunization schedule, COVID-19 pandemic, HPV vaccines

## Abstract

**Objective:** To analyze the number of applied HPV vaccine doses before (from April 2019 to March 2020) and after (from April 2020 to September 2020) social distancing measures in response to the COVID-19 pandemic in states and regions of Brazil.

**Methods:** Ecological time-series study, using data from the Brazilian National Immunization Program (PNI). Using the Mann-Whitney test, we evaluated the difference between the median number of applied doses during the periods April 2019 to March 2020 and from April 2020 to September 2020. Spatial analysis identified clusters with a high or low percentage reduction in the median applied doses. Prais-Winsten regression models identified temporal trends in the applieddoses from both periods.

**Results:** There was a significant reduction in the median HPV vaccine doses applied, formation of spatial clusters and, after a sharp drop in the number of applieddoses during the months following social distancing. There was a tendency to increase the applied vaccines doses.

**Conclusion:** The COVID-19 pandemic resulted in reduction of the number of HPV vaccine doses applied as a possible effect of restrictive measures caused by the pandemic.

## Introduction

Severe Acute Respiratory Syndrome Coronavirus-2 (SARS-CoV-2), the etiological agent of the disease caused by the Coronavirus 2019 (COVID-19), was first identified in Brazil in February 2020 and, in March of that same year, strategies were adopted to mitigate the risk of virus transmission through the use of masks, hand hygiene and social distancing [1]. In order to reduce rates of hospital admissions and prevent the collapse of the health system, Brazilian states and municipalities closed schools and non-essential businesses, banned events that promoted the gathering of people and, in some municipalities and states, imposed curfews on entire populations [1, 2].

Although national and international health agencies have recommended continuing immunization actions during the COVID-19 pandemic, especially as it is an essential health strategy [3–5], studies point to a reduction in vaccination coverage in adolescents, attributing it to a hesitation by those parents or guardians who fail to seek out vaccination services, due to the risk of infection [4, 6–11].

A study carried out in Pakistan identified a 52.5% reduction in the daily average of the number of vaccines administered during the period of social distancing compared to the period before the pandemic, and the reduction was attributed to both a decrease in demand and the supply of immunization in the country [6]. In England, there was a reduction of 6.7% in the administered doses of the hexavalent vaccineand hepatitis B, after the adoption of social distancing measures when compared to the same period of the previous year [12]. In addition to compromising herd immunity, low vaccination coverage favors the formation of pockets of susceptible individuals in certain areas and allows for the transmission of infections from sick individuals to individuals who have not been adequately immunized, resulting in a public health problem [4, 7, 10, 11].

Among the preventable infectious diseases, cervical cancer stands out, which is the fourth most common type of cancer among women, caused in large part, by 90% of Human Papillomavirus (HPV) types 16 and 18 viruses [13]. In addition to cervical cancer, HPV types 16 and 18 can cause vulvar and vaginal cancer in women, penile cancer in men and oropharyngeal cancer in the oral cavity and anal region in both sexes [14, 15]. Vaccination against HPV, given preferably before the individual begins sexual activity, is more than 93% effective in preventing persistent infections and precancerous lesions caused by HPV [13, 16]. In Brazil, in 2014, the quadrivalent HPV vaccine, which prevents HPV types 6, 11, 16, and 18, began rollout free to the public by the National Immunization Program (PNI) targeting young girls aged 9–13 years, and vaccine application was gradually expanded to include girls, aged 14, and boys, aged 11–14 years [17].

Vaccination against HPV, combined with cervical cancer screening, offered to the female population, has been responsible for the reduction in the incidence of cervical cancer in countries over the last decade [16, 18]. However, recent studies indicate that high-income countries have more effectively adhered to cervical cancer vaccination and screening strategies than low- and middle-income countries, which reinforces the global inequalities of access to prevention of cervical cancer strategies [16, 19]. In this sense, considering that it is foreseeable that during the period of the declared health emergency, due to the COVID-19 pandemic, population access to services that offer vaccination in Brazil have become less frequent [11, 20] during the short and long term periods. In the short term, there may be a reduction in the number of applieddoses of all vaccines, including the HPV vaccine. This scenario can compromise vaccination coverage and increase the number of individuals who will not have access to the primary cervical cancer prevention strategy at certain Brazilian locations.

In Brazil, it is worth noting that the recommended vaccinecoverage targeting children and adolescents is not homogeneous, and the formation of pockets of individuals who are not adequately immunized and, therefore, susceptible to specific infectious diseases in some locations, is commonly reported [21]. In this regard, studies that adopted spatial analysis to highlight where pockets of susceptible individuals are located, can support public policies and health strategies to improve immunization indicators, a goal included in the 2030 Agenda of the United Nations Sustainable Development Goals [22]. So far, few studies have evaluated the impact of the COVID-19 pandemic on the vaccination of children and adolescents.

Thus, the aim of this study was to analyze the number of doses of the HPV vaccine applied before and after the start of social distancing measures in response to the COVID-19 pandemic in Brazil.

## Methods

### Study Design and Data Collection

This was an ecological time-series study, with data from April 2019 to September 2020, collected from the Information System of the National Brazilian Immunization Program (SI-PNI), available at: http://sipni.datasus.gov.br/. The SI-PNI has been gradually implemented in Brazil since 2012, however, since the 1990s, appliedvaccinesdoses in Brazil have been registered and monitored monthly [23]. For data extraction, the monthlyapplieddoses to the target population over the period were tabulated, which corresponded to two doses of the vaccine given to young girls, aged 9 to 14 and in young boys, aged 11 to 14, totaling 4,794,787 doses.

### Statistical Analysis

First, the applied vaccinedoses of HPV before (April 2019 to March 2020) and after (April to September 2020) the start of social distancing measures enacted in Brazil in the 27 States and the Federal District were included. Next, the differences between the median number of applieddoses, before and after the social distancing measures, were assessed using the Mann-Whitney U test, considering the interquartile range (IIQ) and a significance level of 5%. The percentage of variation of the median number of applieddoses was estimated using the equation:

[(median number of doses applied before social distancing measures-median number of doses applied after social distancing measures)/median number of doses applied before social distancing measures ×100]

To analyze the presence of spatial clusters formed by regions with a high or low percentage reduction in the median of doses applied, before and after social distancing measures in the 27 States and the Federal District, a spatial association was estimated using theGlobal Moran’s Index (Moran’s I). This index estimates the spatial autocorrelation, being positive, when Moran’s I > 1, no correlation, when Moran’s I = 0, or an inverse correlation when Moran’s I < 1 [24]. For interpretation of the strength of the spatial correlation, Moran’s I was classified as weak (<0.3), moderate (0.3–0.7) or strong (>0.7) [24]. TerraView software, version 4.1.0, was used for these analyzes from the Local Indicators of Spatial Association (LISA). Mapbox maps were elaborated from a cartographic data base, which present the States and Federal District line limits for representing the distribution of the percentage reduction of the median applieddoses before and after social distancing measures. The clusters were classified as High-High (high percentage of reduction surrounded by high percentage of reduction) or Low-Low (low percentage of reduction surrounded by low percentage of reduction).

To verify the effect of social distancing measures on time trends of the number of appliedvaccine doses, this study considered an interrupted time series (before and after the start of social distancing measures). The time trends of the absolute number of doses applied in the five Regions of Brazil (North, Northeast, South, Southeast, and Midwest) were calculated during each of the studied months. Prais-Winsten regression models were used to identify significant trends in the temporal variation of the number of applieddoses. This model is based on linear regression analysis and aims to correct the effect of autocorrelation, which is recommended fortemporal trend studies [25]. In this analysis, the variable of interest was the absolute number of doses for each month and, as an explanatory variable, the reference month. The existence of a significant trend was considered when the slope (β) of the model was found to be different from zero and a *p*-value less than or equal to 0.05 (*p* < 0.05). The positive β indicates an increase in the variation of the absolute monthly number of applieddoses during the period and a negative β indicates a reduction. When no statistically significant difference was identified (*p* ≥ 0.05), the trend was considered to be stationary. The accuracy of the model was expressed by the coefficient of determination (R2). To verify the existence of autocorrelation in the series, the Durbin-Watson test was applied for the entire study period. Time series trend analysis were performed using the Software for Statistics and Data Science (Stata) program, version 14.

For all analysis, the level of statistical significance was set at 5% (0.05). The Stata (version 16.0) was used for these analyzes.

### Ethical Aspects

Due to the nature of this study, by using free access data, it was not necessary to submitthe project to the Research Ethics Committee, in accordance with resolution 466/2012 of the National Health Council [26].

## Results

From April 2019 to September 2020, 4,794,787 doses of the vaccine against HPV were applied throughout Brazil, with 3,660,334 (76.34%) in the period from April 2019 to March 2020 and 1,134,453 (23, 66%) in the period from April 2020 to September 2020. In the period prior to the recommendations for social distancing, the median number of applieddoses was 309,220 (IIQ: 269,382–352,761) and during the duration of these recommendations, the median number of applieddoses was 189,994 (IIQ: 167,705–262,311), equivalent to a reduction of 38.55% (*p* = 0.006). Three of the five Regions in the country (North, Northeast and South) showed a statistically significant reduction in the median of applieddoses during the duration of the social distancing measures. Among the 27 States and the Federal District, in 16 of them, the reduction in applied vaccinedoses of HPV was statistically significant, ranging from 21.44% in Mato Grosso (*p* = 0.044) to 63.16% in Amapá (*p* = 0.002) (Table 1).

**TABLE 1:** Median and percentage variation of the median number of applied doses of HPV vaccine in Brazil before and during social distancing measures. The National Immunization Program, April 2019 to September 2020.

States and regions	Median (interquartile range)			
	Apr-19 to Mar-20	Apr-20 to Sep-20	Variation (%)	p*
Brazil	309,220 (269,382–352,761)	189,994 (167,705–262,311)	−38.55	**0.006**
North	3,356 (1,258–14,002)	2,143 (584–6,970)	−36.14	**0.015**
Acre	633 (529–856)	281 (240–446)	−55.60	**0.004**
Amapá	1,344 (1,087–1,852)	495 (108–1,087)	−63.16	**0.002**
Amazonas	11,325 (7,795–14,284)	6,962 (2,788–11,441)	−38.52	**0.044**
Pará	14,673 (12,732–22,634)	9,808 (4,974–12,734)	−33.15	**0.008**
Rondônia	3,096 (2,914–4,477)	2,320 (1,934–3,038)	−25.06	**0.020**
Roraima	1,255 (995–1,369)	746 (501–1,247)	−40.55	**0.035**
Tocantins	3,980 (2,485–4,699)	2,109 (2,080–3,430)	−47.01	0.085
Northeast	9,027 (4,859–17,155)	5,442 (3,312–12,949)	−39.71	**0.006**
Alagoas	4,040 (3,301–4,609)	3,824 (2,274–4,636)	−5.34	0.536
Bahia	30,029 (19,889–32,995)	16,187 (13,824–18,443)	−46.09	**0.011**
Ceará	14,182 (11,335–16,094)	12,067 (5,678–14,782)	−14.91	0.246
Maranhão	10,385 (9,185–18,912)	8,040 (4,665–8,579)	−22.58	**0.008**
Paraíba	5,004 (3,975–6,829)	4,195 (2,586–5,318)	−16.16	0.179
Pernambuco	13,996 (10,532–16,049)	12,906 (10,089–13,033)	−7.78	0.479
Piauí	5,739 (5,178–7,119)	3,598 (3,022–5,335)	−37.30	**0.035**
Rio Grande do Norte	4,911 (3,642–5,716)	3,642 (2,873–4,225)	−25.83	0.104
Sergipe	4,202 (3,025–5,793)	2,949 (2,143–3,240)	−29.81	**0.044**
Midwest	10,649 (5,242–29,289)	6,988 (3,811–22,163)	−34.37	0.136
Distrito Federal	5,274 (4,125–6,739)	3,793 (3,033–5,365)	−28.08	0.126
Goiás	8,972 (7,697–12,201)	6,988 (5,556–9,402)	−22.11	0.085
Mato Grosso	39,323 (32,579–48,844)	30,889 (24,131–38,796)	−21.44	**0.044**
Mato Grosso do Sul	4,001 (3,343–4,920)	3,811 (3,400–4,327)	−4.74	0.724
Southeast	16,246 (9,001–95,948)	14,543 (5,123–79,336)	−10.48	0.061
Espírito Santo	9,001 (7,010–11,553)	5,260 (4,014–6,790)	−41.56	**0.004**
Minas Gerais	8,196 (5,630–9,583)	4,520 (3,748–7,848)	−44.85	**0.008**
Rio Janeiro	16,246 (13,280–17,498)	14,543 (8,531–19,053)	−10.48	0.375
São Paulo	85,210 (78,285–102,915)	53,702 (44,200–80,828)	−36.97	0.056
South	25,914 (15,549–47,152)	12,269 (10,295–23,495)	−52.68	**0.000**
Paraná	26,441 (25,418–34,585)	13,712 (11,004–17,660)	−48.14	**0.000**
Santa Catarina	14,673 (11,230–24,213)	8,347 (7,926–10,883)	−43.11	**0.003**
Rio Grande do Sul	18,179 (13,275–22,034)	11,735 (10,632–14,592)	−35.44	**0.015**

Note: *p* = percentile *Mann-Whitney test (difference between medians). *p*-values marked with bold indicate statistically significant *p*-values.

**FIGURE 1:**
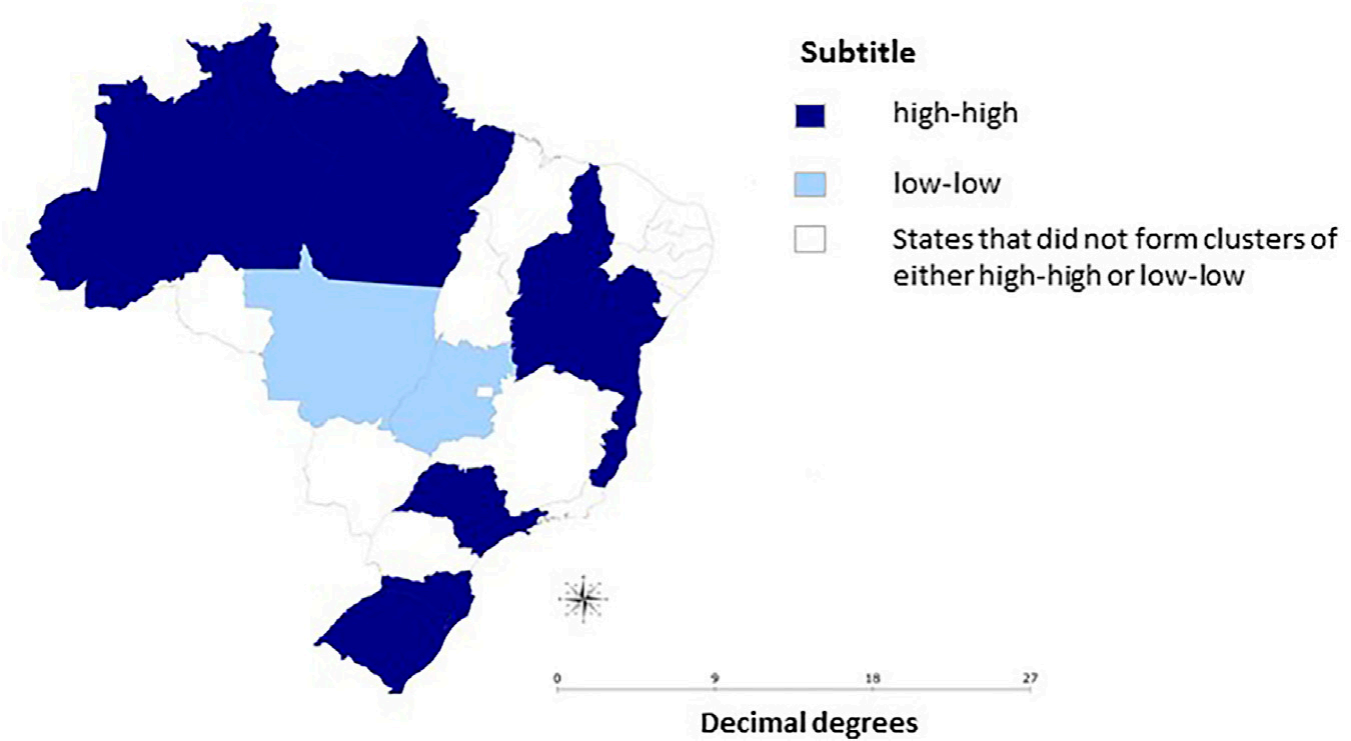
Mapbox of the percentage reduction of the median number of doses applied before and after the measures of social distancing in Brazil. The National Immunization Program, April 2019 to September 2020.

Moderate spatial autocorrelation (Moran’s I = 0.39; *p* = 0.003) of the percentage reduction in the median of applied doses was identified. Low-Low spatial cluster (reduction between 21.44% and 22.11%) was formed by the States of Mato Grosso and Goiás. High-High spatial clusters (reduction between 33.15% and 63.16%) were formed by the States of Acre, Amapá, Amazonas, Pará, Roraima, Bahia, Piauí, Espírito Santo, São Paulo, Santa Catarina, and Rio Grande do Sul (Figure 1).

**FIGURE 2:**
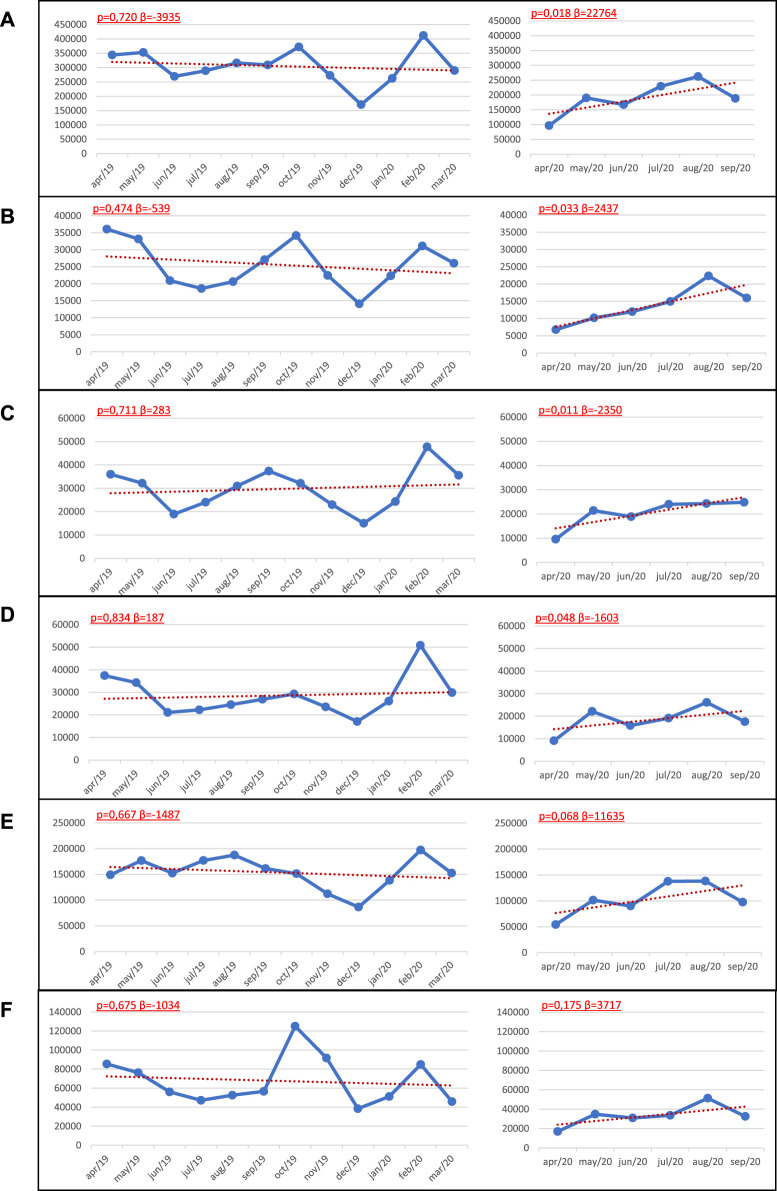
Trends of the number of doses of HPV vaccine applied in **(A)** Brazil and regions **(B)** North **(C)** Northeast **(D)** Southeast **(E)** Midwest, and **(F)** South. National Immunization Program, April 2019 to September 2020. Note: *Prais-Winsten* regression model *p* = valor, β = slope.

Figure 2 presents the time series of the number of applieddoses for the HPV vaccine in Brazil and macro-regions. A noticable drop in doses in March and April 2020 was observed during a period in which social distancing measures were initiated and which were more intense. Begining in May 2020, there was a change in the trend of the number of applieddoses, from stationary to increasing in Brazil, the North, Northeast and Midwest regions (*p* < 0.05) (Figure 2).

## Discussion

This study demonstrates that the COVID-19 pandemic resulted in a reduction in the number of HPV appliedvaccine doses in Brazil, as a possible effect of restrictive actions againstthe pandemic. With the exception of the Midwest and Southeast Regions, the other three Regions presented a significant reduction in the median doses of HPV vaccine applied during the duration of the recommendations for social distancing. Spatial clusters of the High-High type were formed by States that presented a percentage reduction in the median applieddoses of HPV vaccine between 33.15% and 63.16% and were mainly located in the Northern Region. The trend analysis, in turn, revealed an abrupt drop in doses between March and April 2020 and, starting in April, a trend towards an increase in the number of applieddoses in Brazil, North, Northeast and Midwest regions (*p* < 0.05).

National and international studies have attributed the reduction of the population’s demand for health services with a consequent drop in vaccination coverage to the restrictive measures adopted by governments during the COVID-19 pandemic [4, 7, 8]. However, it is worth noting that there has been an observed trend of declining applieddoses of vaccines in Brazil over the past 2 decades [8], especially those immunobiologicals recommended during early childhood [27, 28]. Contextual and individual factorsthat are commonly cited by studies that investigate the reasons for the reduction of vaccine coverage include a precariousness of the Brazilian National Healthcare System (SUS), social and cultural aspects that compromise vaccine acceptance, limited periods of routine schedules for receiving several vacines offered by the National Immunization Program, anti-vaccine movements, and inconsistencies of the availability of immunobiologicals supplied bythe Primary Healthcare System [8, 29]. The findings of our study demonstrate that, added to the historical reduction in the coverage of the HPV vaccine in Brazil [17], there was a sharp drop in the number of applieddoses of the HPV vaccine during the first 6 months, when compared to the previous period of COVID-19 pandemic.

During the period prior to the start of social isolation measures, the number of administeredHPV vaccine doses was lower than observedin other months from the same period (April 2019 to March 2020). The reduction in the number of doses in these months can be attributed to the interruption of immunization actions that are commonly developed in schools during the school term [30], impacting on the recommendednumber of vaccinesdoses for children and adolescents. Otherwise, in the United States, there is a trend towards an increase in the recommendednumber of vaccines doses for children and adolescents during the summer vacation, a fact that can be explained by the increased demand by parents for immunization services in order to normalize their children’s vaccination status, since proof of immunization is a mandatory requirement for children and adolescents to enroll at schools [31].

The scenario of statistically significant reduction in the median of doses applied during social distancing measures, added to the drop in vaccination coverage rates in recent years, point to a problem for collective immunity and the risk of resurgence of diseases that are considered under control or eradicated [28, 32]. Furthermore, it is worth highlighting the regional inequalities in vaccination coverage in Brazil that favor the formation of pockets of susceptible individuals [21, 27, 28, 33]. A study that evaluated the coverage of the HPV vaccine in Brazil, from 2013 to 2017, identified areas with low vaccination coverage in the North of the country [34]. Considering that HPV vaccination is the main strategy for the prevention of cervical cancer [16, 18], a low vaccination coverage against HPV [17] aggravated bysocial distancing measures, as demonstrated by this study, may, in the medium and long term, contribute to an increase in cervical cancer rates in the North of Brazil.

These regional inequalities in vaccination coverage, as pointed out in recent studies [17, 27], can be attributed, in part, to historical differences in health sector investments that have contributed to the precariousness of the structure of primary healthcare services in the North and Northeast Regions when compared to other Brazilian Regions [29, 35–37]. The increase in the number of health facilities over the last 30 years, accompanied by an increase in population coverage by the National Family Healthcare System and primary healthcare teams, has contributed inexpanded population access to these services. But still, inequalities in the structure of health services still persist.

The most precariousinfrastructure conditions and access to services are located in the North and Northeast regions of the country [35, 37]. Considering that the National Immunization Program offersfree of charge immunization to its population, spearheaded by the country’s primary healthcare services, the persistent precariousness of these public services influences the availability of immunobiologicals and, consequently, compromises the vaccination situation of population groups residing in regions covered by these same services [29].

Each one of the Brazilian states implemented strict, mitigation measures for the prevention and control of the COVID-19 pandemic from the second half of March 2020 [1]. These measures may have contributed to the sharp reduction in the number of applied doses of the vaccine against HPV from April to May 2020. However, from May 2020, there was an observed change in the trend of the number of applied doses of the HPV vaccine, from stationary to increasing. This change in trend can be explained by the flexibility of social distancing measures that were initially adopted [36], which favored the circulation of people and an increase in demand for immunization services [11], resulting in an increasing trend of the number of applied doses of the HPV vaccine throughout the country. However, in spite of the increased trend in the number of applied doses of HPV vaccine, following the adoption of social mitigation measures, there was a significant percentage reduction in the median dose of HPV vaccine observed in the North, Northeast and South regions when compared to the period prior to social distancing (*p* < 0.05).

It is worth noting that the collapse of healthcare services in some states in the North and Northeast regions, due to the increased demand for beds for the hospitalization of patients with COVID-19, may have contributed to the reduction in the population’s demand for immunization services in these regions [38, 39]. In addition, strategies to contain the pandemic in the states and regions of Brazil were not uniform, which may explain the percentage variations in the median applieddoses of the HPV vaccine, ranging from 21.44% in the State of Mato Grosso (*p* = 0.044) to 63.16% in Amapá (*p* = 0.002). While some locations, in response to the epidemic phase of the accelerating increase in number of cases and deaths by COVID-19, adopted social distancing, others resorted to the strategy of total confinement, suspending all non-essential activities and limiting the circulation of people [36, 40].

In the Southern region, the States of Santa Catarina and Rio Grande do Sul adopted early social distancing measures and, for several weeks, the Southern regional states demonstrated the best isolation index in Brazil [1]. The early adoption of the social isolation strategy, associated with the population’s adherence to the instituted measures, may have contributed to the reduction in 52.68% ofthe median doses of HPV vaccine in this Region, corresponding to the greatest percentage reduction in doses of HPV vaccine between the Brazilian regions (*p* = 0.000). In Brazil, after more than a year since the first reported case of COVID-19, we are still in the epidemic phase of acceleration of the number of cases and deaths, with less than 4.6% of the population immunized against COVID-19 at the time of writing [41] and with predictions models that point to the long-term practice of social distancing strategies [42]. In this scenario, it is imperative to adopt health strategies and policies that ensure the population’s access to immunization against HPV and other infectious diseases, thus avoidingthe consequences of an overlap of COVID-19 cases and deaths with other infectious diseases.

### Limitations

This study presented intrinsic limitations to studies using secondary data, highlighting the impossibility of establishing causal inferences, since the data were collected during a single moment. In addition, some variables that were not collected or are omitted from the database, could have favored our understanding of the object of this research. However, but there was a lack of control by the researchers regarding the standardization and quality of data recorded by the SI-PNI. The SI-PNI is considered a powerful tool as a source of data regarding immunizations of the Brazilian population, and although the system may present some draw backs, as pointed out, it has a solid, technical bases, with more than 40 years of existence, contributing to support monitoring and decision-making at the municipal, state and federal levels [43, 44]. The SI-PNI is useful in the fulfillment of its mission, being decisive both in the control and validating the identification of groups susceptible to vaccine-preventable diseases through individualized data and the management of preventive actions throughout Brazilian regions [45]. A study that evaluated the SI-PNI reported that the system maintains sensitive criteria, managing to capture a satisfactory percentage of vaccination of the population [43]. In addition, the SI-PNI is coordinated by the Ministry of Health and has stringent regulations for control, adherence and standard fulfillment targets [43]. Therefore, the data reported here are well accepted and are close to reality for this country.

### Conclusion

The COVID-19 pandemic resulted in a reduction in the number of applieddoses of the HPV vaccine as a possible effect of the restrictive actions by government health officials in an attempt to reduce the spread of the virus. To our knowledge, this was the first study of its kind in the country to consider the HPV vaccine. We believe that the results of this study may support public health policies to guarantee strategies for immunization against HPV in Brazil even during the current epidemic crises, in which there has been an increase in the number of cases of COVID-19 throughout the country.
